# A Case of Psoas Abscess Diagnosed With Oral Bacteria as the Causative Pathogen

**DOI:** 10.7759/cureus.97584

**Published:** 2025-11-23

**Authors:** Koki Umemori, Kyoichi Obata, Mayumi Yao, Koji Fujita, Soichiro Ibaragi

**Affiliations:** 1 Oral and Maxillofacial Surgery, Okayama University Graduate School of Medicine, Okayama, JPN; 2 Dentistry and Dental Surgery, Tsuyama Central Hospital, Tsuyama, JPN; 3 General Internal Medicine and Infectious Diseases, Tsuyama Central Hospital, Tsuyama, JPN

**Keywords:** hematogenous spread, oral diseases, oral health care, pseudomonas aeruginosa, psoas muscle abscess, streptococcus oralis

## Abstract

We report a rare case of a psoas abscess in an 87-year-old woman, in which oral commensal bacteria may have disseminated hematogenously from a chronic oral infection site and served as the causative pathogens. The patient presented with persistent left buttock pain, fever, and swelling, and imaging revealed a fracture of the left iliac bone with an associated psoas abscess. Bacterial cultures identified *Streptococcus oralis* and *Pseudomonas aeruginosa*. Her symptoms improved following antibiotic therapy and CT-guided drainage. Although the presence of *P. aeruginosa *in the oral cavity is generally considered transient, it has been isolated from the oral cavities of elderly and immunocompromised individuals. In the absence of lacerations or other direct portals of entry, and considering the identification of both pathogens, the oral cavity was regarded as the most likely source of infection. This case highlights the importance of correlating culture results with the most probable source of infection to improve the prognosis of systemic infections.

## Introduction

A psoas abscess is an infection of the psoas muscle that leads to localized abscess formation [1]. It is classified as primary, which occurs through hematogenous or lymphatic spread of bacteria without any infectious source in adjacent organs, and secondary, which results from the direct extension of infection from nearby organs [2]. More than half of all cases are secondary, while primary abscesses account for approximately one-third [3]. Trauma may act as a precipitating factor; however, prolonged sitting or recumbency can also place continuous pressure on the gluteal region, causing local tissue injury and increasing susceptibility to infection [4]. Consequently, *Staphylococcus aureus*, a common skin commensal, is identified as the causative organism in approximately 42.9% of reported cases [5]. Other risk factors include diabetes mellitus, liver cirrhosis, chronic renal failure, the use of steroids or immunosuppressants, and HIV infection [6]. The incidence of psoas abscess has been reported as 1.21 cases per 100,000 population in a domestic report from Japan, whereas in the United Kingdom, an incidence rate of 0.4 cases per 100,000 population has been documented [7,8]. Although improvements in nutrition and the development of antibiotics have made this disease relatively uncommon, its frequency has been increasing in recent years, particularly among elderly patients [3]. If left untreated, the mortality rate can approach 100% [6]. It most frequently affects men in their 60s and typically presents with fever, localized pain, and psoas muscle abnormalities. However, these symptoms may be atypical or less pronounced in elderly individuals [3]. The most common causative pathogen is *Staphylococcus aureus* [5]. However, cases caused by *Streptococcus oralis* remain extremely rare. Here, we describe a rare case of a psoas abscess caused by the oral commensal bacterium *S. oralis*.

## Case presentation

An 87-year-old woman presented with persistent left buttock pain and recurrent fevers (~38°C) that began after she fell at home one month earlier and sustained a direct blow to her left buttock. She initially visited a local clinic, where she was prescribed antibiotics and analgesics; however, her symptoms did not improve. She subsequently sought care at another medical facility, where a computed tomography (CT) scan revealed abscess formation around the left iliac bone. Consequently, she was referred to the Department of Orthopedic Surgery at Tsuyama Chuo Hospital for further evaluation.

On admission, her vital signs were as follows: body temperature 37.2°C, blood pressure 138/72 mmHg, heart rate 72 bpm, and SpO₂ 99% on room air. Physical examination revealed tenderness extending from the left buttock to the left back, along with marked edematous swelling of the left lower extremity. No lacerations were observed on the skin overlying the injured area. Her medical history was notable for hypertension, for which she was taking antihypertensive medication. She had no history of immunosuppressive agents. However, considering her advanced age, age-related immune decline could not be excluded.

Laboratory tests showed a white blood cell (WBC) count of 6,600/μL, an absolute neutrophil count of 5,630/μL, and a C-reactive protein (CRP) level of 16.45 mg/dL (Table 1).

**Table 1:** Laboratory findings at presentation WBC: white blood cell; ANC: absolute neutrophil count; CRP: C-reactive protein

Test	Result	Reference Range
WBC( /μL)	6,600	3,300-8,600
ANC (/μL)	5,630	1,650-6,020
CRP (mg/dL)	16.45	0.00-0.14

**Figure 1:**
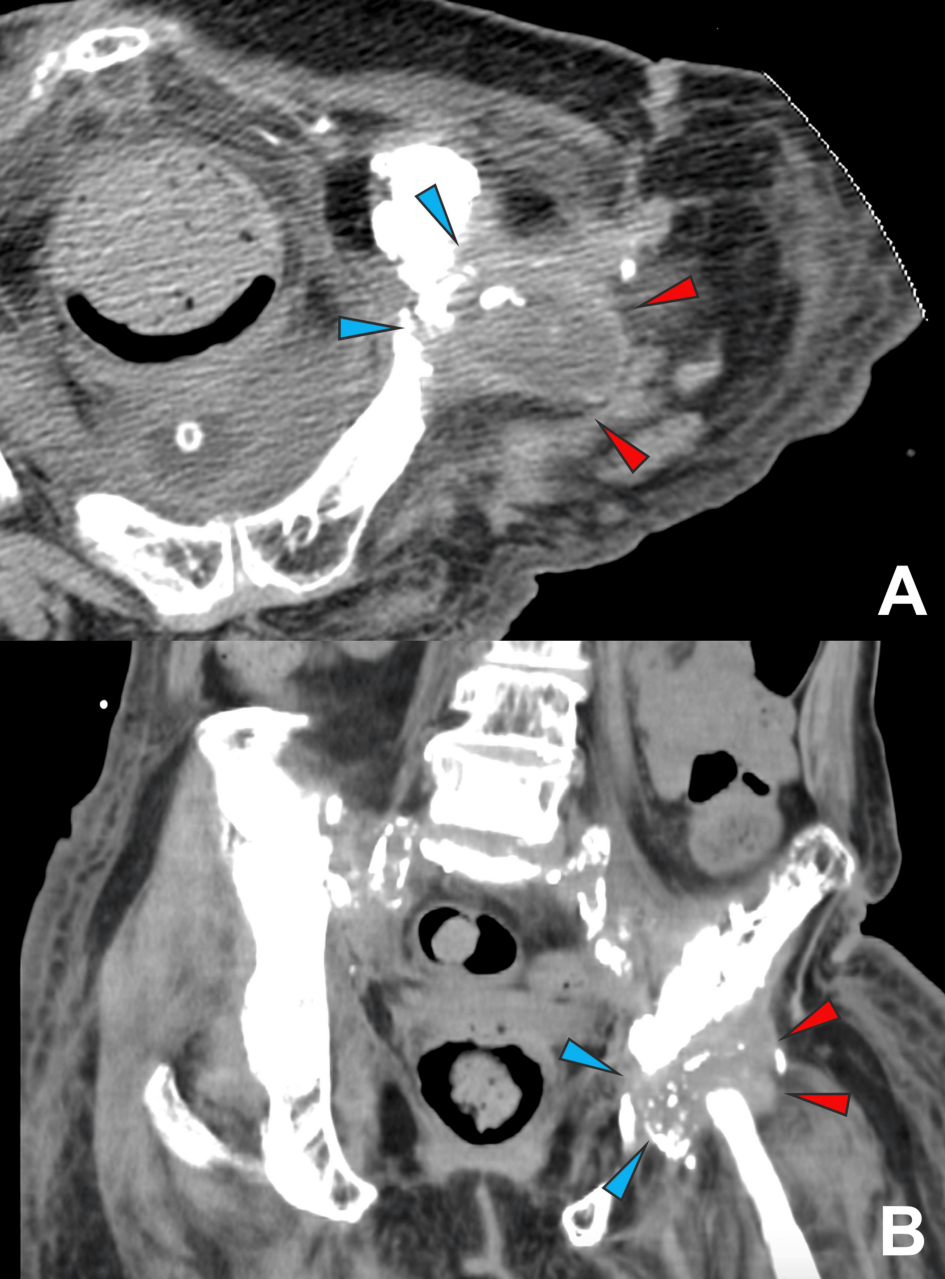
Contrast-enhanced lumbar CT images A, B: blue arrowheads indicate osteolytic changes in the left iliac bone, and red arrowheads indicate abscess formation.

Procalcitonin was also positive, and blood cultures obtained at the referring hospital yielded α-hemolytic *Streptococcus* species. CT imaging demonstrated a fracture of the left iliac bone accompanied by fluid accumulation in the surrounding soft tissues. No infectious foci were observed in the surrounding organs (Figure 1).

Although *Streptococcus* species are well-recognized causative pathogens of infective endocarditis, echocardiographic evaluation revealed no evidence of valvular involvement in this patient. Based on these findings, the patient was diagnosed with a left iliac bone fracture and a left psoas abscess. Intravenous cefazolin (CEZ) was initiated on the first hospital day, and CT-guided percutaneous drainage was performed. Bacterial culture of the drainage fluid identified *Pseudomonas aeruginosa* and *Streptococcus oralis*, an oral commensal bacterium. Based on these findings, the antibiotic regimen was changed from CEZ to piperacillin to target *P. aeruginosa*; however, due to a drug eruption, it was subsequently switched to cefepime. In light of the identification of *S. oralis*, the patient was referred to the Department of Dentistry and Oral Surgery on hospital day 6. By hospital day 13, follow-up CT confirmed resolution of the psoas abscess, and the drainage catheter was removed.

**Figure 2:**
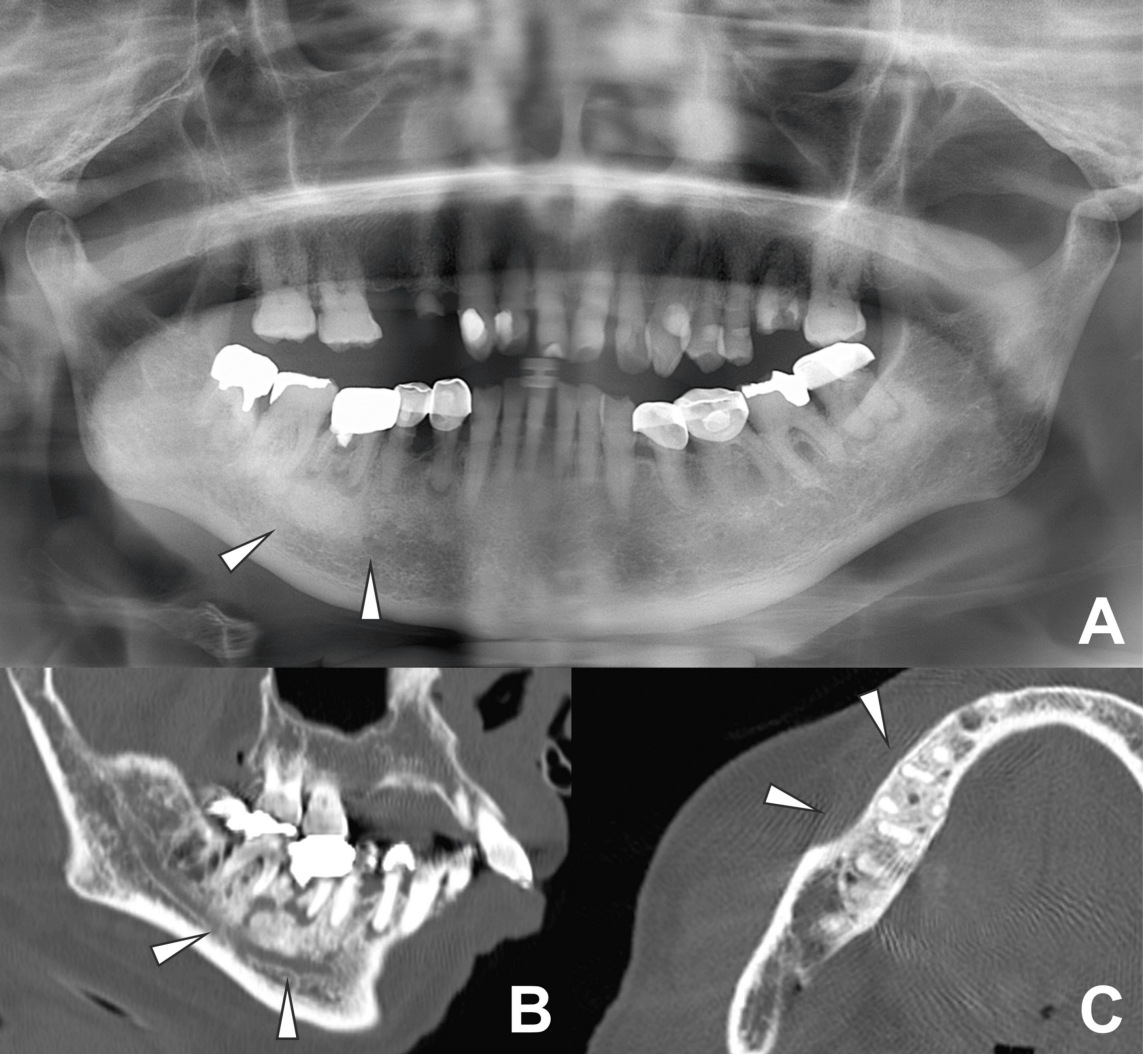
Panoramic radiograph and head CT images A: panoramic radiograph indicates sclerosis of the right mandibular bone (arrowheads); B, C: head CT images indicate a periapical lesion at the root of the right molar, with significant surrounding sclerosis (arrowheads).

Oral examination revealed poor oral hygiene, generalized gingival swelling, and multiple periapical lesions. Panoramic radiography and CT imaging revealed findings suggestive of osteomyelitis of the right mandible, periapical periodontitis of the right maxillary first premolar, and cystic lesions around the left maxillary canine and first molar regions (Figures 2A-2C).

A multidisciplinary conference comprising orthopedic surgeons, infectious disease specialists, oral and maxillofacial surgeons, and radiologists was conducted, and as there were no lacerations on the skin over the injured area, a transcutaneous route of infection was considered unlikely, and the patient’s condition was thought to be explained by the following two possible mechanisms: bacteremia originating from oral infections and hematogenous spread of oral bacteria leading to the psoas abscess.

**Figure 3:**
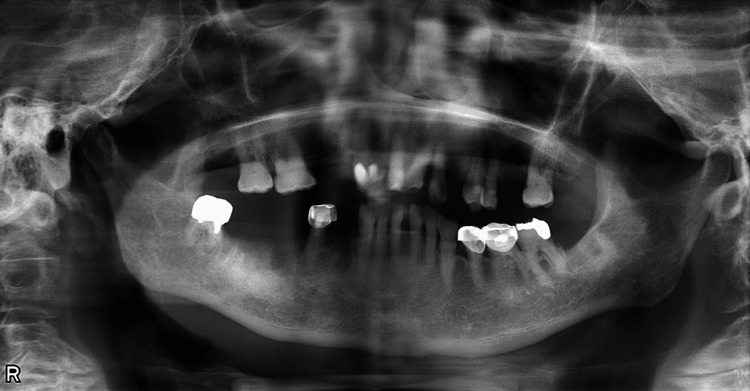
Post-treatment panoramic radiograph The oral focus of infection was successfully removed.

Drainage alone was considered insufficient, and removal of the oral infectious foci was prioritized. Initial oral cleaning was performed by a dental hygienist prior to the removal of the infectious foci. On hospital day 13, extraction of the right maxillary first premolar, left maxillary canine, and first molar, along with cystectomy, was performed. On hospital day 18, extraction of the right mandibular premolars and molars, along with saucerization, was carried out (Figure 3). 

**Figure 4:**
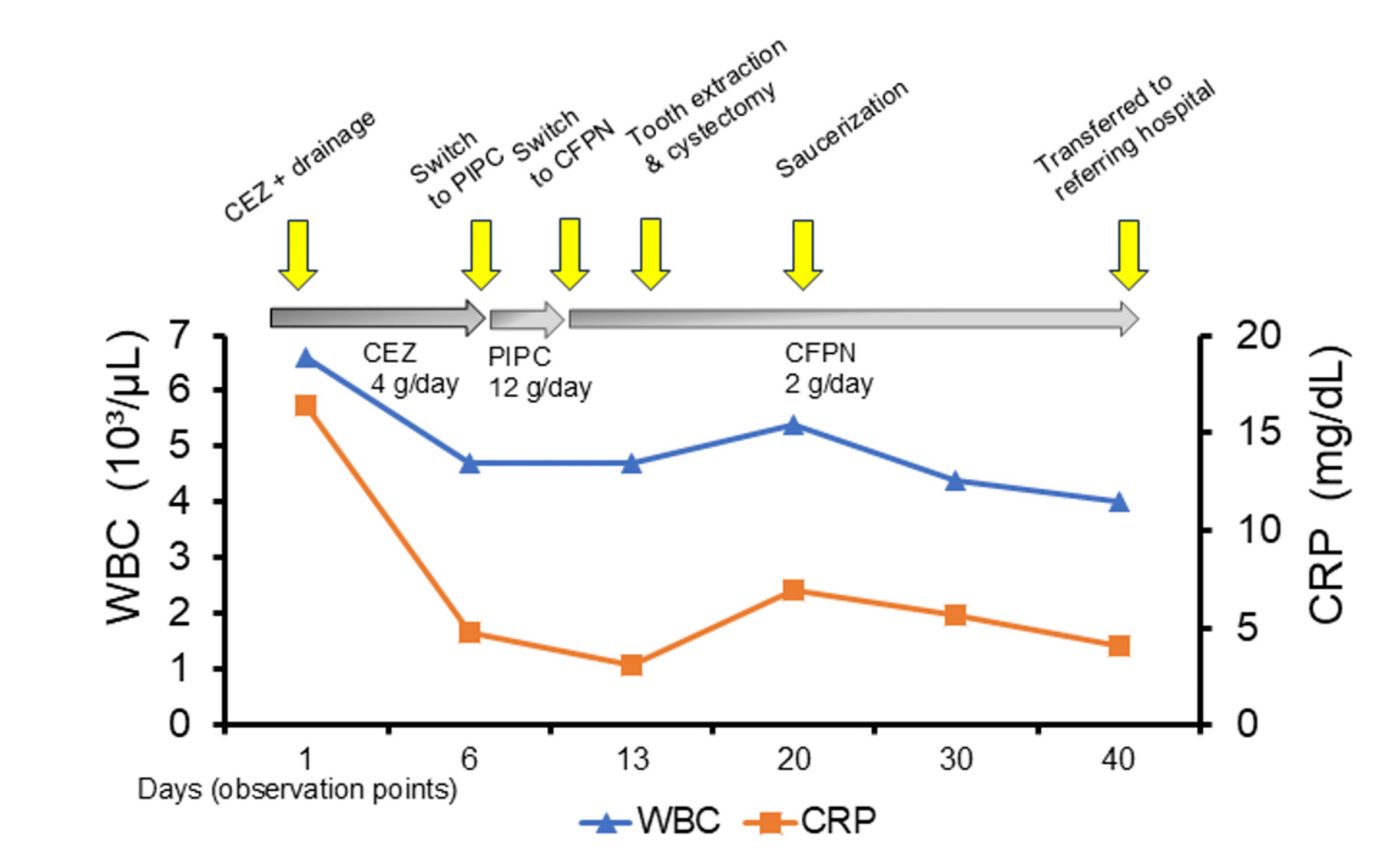
Time course of WBC and CRP levels from the initial presentation The line graph indicates changes in WBC and CRP levels, demonstrating a steady decline following treatment. CRP: C-reactive protein; CEZ: cefazolin; PIPC: piperacillin; CFPN: cefepime

Histopathological examination of the excised cystic lesion revealed a radicular cyst. Neither *S. oralis* nor *P. aeruginosa* was isolated from the surgical specimens of the oral lesions, most likely because systemic antibiotics had already been administered for 18 days prior to the dental procedures and specimen collection. Following these procedures, the patient’s WBC count and CRP levels steadily decreased. Complete resolution of the abscess and lumbar symptoms was achieved, and she was transferred back to the referring institution on hospital day 40 (Figure 4).

## Discussion

*S. oralis* is catalase-negative, nonmotile, nonsporing, facultatively anaerobic, and exhibits α-hemolysis on blood agar [9]. *S. oralis* is a commensal organism widely distributed in the oral cavity, pharynx, nasal cavity, gastrointestinal tract, and genitourinary tract [10]. In the oral environment, it is one of the most abundant species and acts as an early colonizer in dental plaque formation [11].

**Figure 5:**
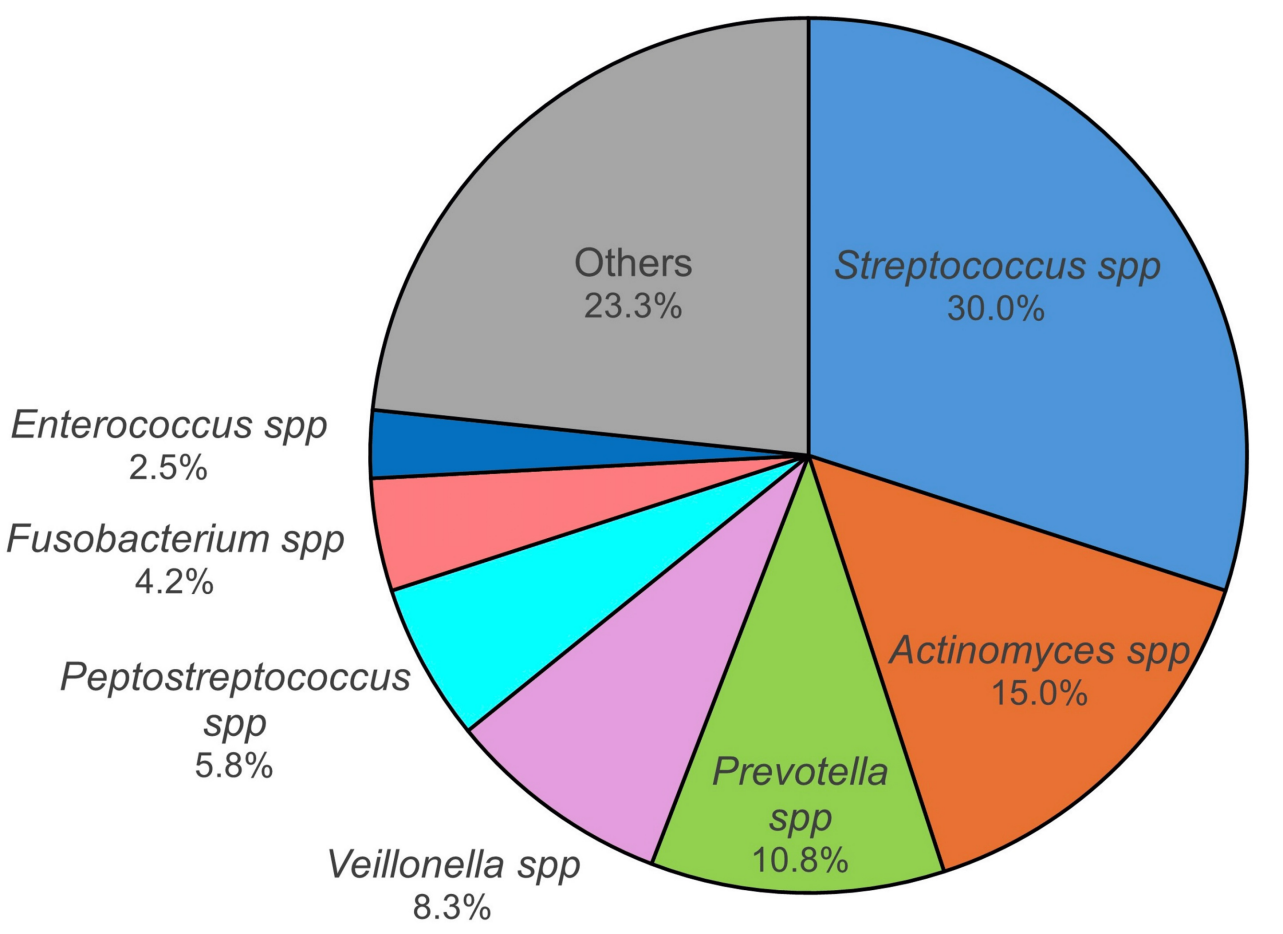
Oral bacteria associated with bacteremia after oral surgery *Streptococcus* species, including *S. oralis*, are the most frequently detected bacteria associated with bacteremia following oral surgical procedures.

The bacterial species responsible for bacteremia following oral surgical procedures are shown in Figure 5. *Streptococcus *species are the most frequently detected, accounting for approximately 30% of all cases, followed by *Actinomyces* (15%) and *Prevotella* species (10.8%) [12]. Invasive dental interventions such as tooth extraction and scaling, as well as routine oral hygiene practices including tooth brushing and flossing, can cause the translocation of *S. oralis* from the oral cavity into the bloodstream, leading to transient bacteremia [10]. Previous studies have reported that the incidence of bacteremia after tooth extraction ranges from 43.1% to 100%, with viridans group streptococci being the most frequently isolated organisms [12]. In cohort studies of viridans group streptococcal bacteremia, *S. oralis* has been identified as the second most prevalent pathogen after *Streptococcus mitis* [11]. Notably, *S. oralis* has been reported as the most common causative species of streptococcal infective endocarditis, accounting for 37.8% of cases [13-15]. These findings indicate that oral procedures and chronic oral infections can serve as potential sources of bacteremia.

This may be attributed to its ability to proliferate in plasma and thrombotic vegetations, as well as to specific virulence factors including serine-rich repeat proteins, adhesion to sialic acid A, and neuraminidase A, all of which contribute to platelet aggregation and endothelial adherence [14,16-19]. These virulence factors may not only facilitate colonization and persistence in the bloodstream but also enable dissemination to distant organs. Oral bacteria are known to be associated with abscess formation in various organs, and cases in which *S. oralis* has been identified as the causative pathogen of abscesses in other organs are summarized in Table 2 [20-25]. However, to the best of our knowledge, no previous reports have described lumbar muscle abscesses caused by *S. oralis*. One possible explanation is that *S. oralis* tends to localize to highly vascularized sites such as the heart, whereas it may have limited access to poorly perfused areas like the psoas muscle. Furthermore, although this organism exhibits strong adherence to vascular endothelium and cardiac endocardium, it may show relatively low affinity for muscle tissue.

**Table 2:** Reported cases of infections in which Streptococcus oralis was identified as the causative pathogen in distant organs

Case	Author	Year	Sex	Age	Site of Infection	Clinical Diagnosis
1	Zubair SN et al. [20]	2022	M	82	Heart	Infective endocarditis
2	Zhang B et al. [21]	2023	M	66	Kidney	Urinary tract infection
3	Solanki R et al. [22]	2014	F	12	Brain	Brain abscess
4	Itoh N et al. [23]	2022	M	77	Liver	Liver abscess
5	Diop AM et al. [24]	2022	F	64	Heart	Infective endocarditis
6	Chu EC et al. [25]	2022	M	60	Spine	Infectious spondylitis
7	Umemori K et al.	2025	F	87	Posas muscle	Posas abscess

In contrast to oral commensals such as *S. oralis*, *P. aeruginosa* is classified as a non-oral bacterium [26]. It is a Gram-negative rod known for causing lower respiratory tract infections and healthcare-associated infections [26]. This pathogen exhibits strong adherence to both host tissues and abiotic surfaces and demonstrates robust biofilm-forming capabilities [26]. It secretes a variety of virulence factors, including elastase and other extracellular enzymes, which contribute to tissue destruction and immune evasion [26]. Furthermore, *P. aeruginosa* possesses multiple antimicrobial resistance genes, making it intrinsically difficult to eradicate with standard antibiotic regimens [26]. Although its presence in the oral cavity is typically transient, it has been isolated from the periodontal pockets of elderly and immunocompromised individuals, where it may exert pathogenic effects under favorable conditions and potentially contribute to the progression of periodontal disease [26].

In this case, the presence of chronic oral infectious foci and the identification of α-hemolytic *Streptococcus* species (including *S. oralis*) in blood cultures strongly suggested the oral cavity as the primary source of bacteremia. Although *P. aeruginosa* is classified as a non-oral bacterium, it can be detected in the oral cavity of elderly or immunocompromised individuals. In this patient, there was no history of systemic diseases associated with immune dysfunction; however, age-related immune senescence may have contributed to increased susceptibility to infection, and an oral origin could not be completely ruled out. At the same time, invasion from the site of injury could not be entirely excluded, which represents a limitation of this case. The detection of two different pathogens further complicated the interpretation of causality. Although the possibility of polymicrobial infection cannot be excluded, contamination was considered unlikely because both organisms were consistently isolated from the abscess specimen under aseptic conditions. In addition, it is possible that a hematoma had formed at the injury site, which may have facilitated hematogenous dissemination of *S. oralis* and *P. aeruginosa*, thereby promoting infection. However, this also represents another limitation of this case. Neither pathogen was isolated from the oral lesions, most likely due to a reduction in bacterial load from systemic antibiotic administration prior to dental procedures and specimen collection, as well as the inherent sensitivity limitations of culture methods. This case underscores the importance of integrating culture results with clinical findings and patient history when the portal of entry and infection route cannot be definitively determined.

## Conclusions

This case highlights the importance of interpreting culture results in relation to the most probable source of infection. Although trauma-related bacteremia could not be completely excluded based on the clinical course, the detection of *S. oralis* suggested an oral origin of infection. Therefore, this case suggests that, regardless of a history of trauma, when oral commensal bacteria are detected in the presence of chronic oral infections, an oral source of infection should be carefully considered. Furthermore, this case indicates that integrating culture results with clinical background to identify the most probable source of infection may contribute to determining appropriate treatment strategies and improving outcomes in systemic infections.
